# Relationship between physical disability and satisfaction with medical care and health facility infrastructure in Peru

**DOI:** 10.31744/einstein_journal/2026AO1223

**Published:** 2025-12-01

**Authors:** Teodoro Gaspar-Roman, J. Jhonnel Alarco

**Affiliations:** 1 Disability Epidemiology Research Group Universidad Científica del Sur Lima Peru Disability Epidemiology Research Group (EpiDIS), Universidad Científica del Sur, Lima, Peru.

**Keywords:** Persons with disabilities, Vulnerable populations, Patient satisfaction, Office visits, Health services, Architectural accessibility

## Abstract

**Objective:**

To analyze the association of physical disability with medical care and health center infrastructure satisfaction among users of medical offices in Peru in 2016.

**Methods:**

We conducted a cross-sectional analysis of secondary data from the 2016 National Health User Satisfaction Survey (ENSUSALUD). The independent variable was self-reported physical disability and the dependent variables were satisfaction with medical care and health facility infrastructure. Sociodemographic variables and variables related to user satisfaction were included as confounding factors. We conducted multiple linear regression analysis and estimated β coefficients with their corresponding 95% confidence intervals (95%CI). The ENSUSALUD 2016 sample design was accounted for in all calculations.

**Results:**

After adjusting for confounders, individuals with moderate physical disabilities reported an average of 1.96 fewer points (95%CI=-2.95 to -0.97) in satisfaction with medical care compared to that of those without physical disabilities. Similarly, individuals with moderate and severe physical disabilities reported averages of 1.86 (95%CI=-2.75 to -0.96) and 6.40 (95%CI=-9.58 to -3.22) fewer points, respectively, in satisfaction with the healthcare facility infrastructure compared to those of individuals without physical disabilities.

**Conclusion:**

In Peru, people with physical disabilities who use medical offices reported lower satisfaction with medical care and infrastructure of health facilities compared to that of those without physical disabilities.

## Highlights

In Peru, 5.2% of the population has a disability, and among them, 59.2% have physical limitations.

People with disabilities face physical/communication healthcare access barriers.

Barriers can affect satisfaction with care received and facility infrastructure.

## In Brief

Gaspar-Roman et al. conducted a secondary analysis of a national user satisfaction survey. They included 13,814 Peruvians who received care at a health facility in 2016. Mean satisfaction scores for medical care and healthcare facility infrastructure were significantly lower among individuals with physical disabilities. Inclusive healthcare should be promoted in health facilities throughout Peru.

## Graphical abstract

**Figure:**
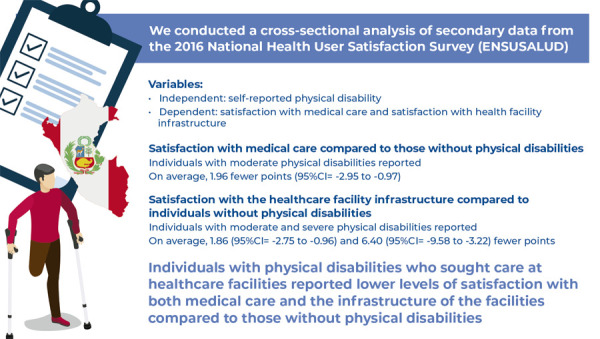

## INTRODUCTION

According to the Pan American Health Organization, individuals with disabilities have physical, mental, intellectual, or sensory impairments that, in the presence of barriers, may hinder their full and effective participation in the society.^( 1 )^ According to the World Report on Disability, approximately 15% of the global population lives with some form of disability.^( 2 )^ In Peru, the first “Encuesta Nacional Especializada Sobre Discapacidad 2012” reported that 5.2% of the population had a disability, with the majority (59.2%) experiencing limitations in the use of their arms and hands or legs and feet.^( 3 )^

Several studies with patients attending healthcare facilities in Peru have reported medical care satisfaction rates of >70%.^( 4 - 6 )^ Low levels of satisfaction have been linked to chronic diseases,^( 5 )^ particularly diabetes mellitus and pulmonary diseases,^( 7 )^ which are often associated with disability

The World Health Organization reports that individuals with disabilities encounter multiple barriers to accessing medical care. These include physical barriers, such as inaccessible or poorly marked environments; communication barriers, such as the absence of sign language interpreters; and economic barriers that restrict access to medications and quality healthcare.^( 8 )^ These obstacles may impact the level of satisfaction of individuals with disabilities when seeking care at healthcare facilities.

Most studies addressing this issue have adopted a qualitative approach.^( 9 , 10 )^ Additionally, studies conducted in some developed countries indicated that individuals with disabilities are more likely to be dissatisfied with their healthcare^( 11 - 13 )^ and that barriers to accessing medical care, the quality of care and service coordination, limited experience of physicians in managing patients with disabilities, and varying levels of disability, are among the primary reasons for this dissatisfaction.^( 12 , 13 )^ However, there is limited evidence on this issue in Latin America, a region characterized by significant health inequities due to the low economic development of its countries. Therefore, gaining insight into how these barriers related to accessibility and healthcare services impact satisfaction levels among individuals with disabilities is crucial.

## OBJECTIVE

We aimed to analyze the association of physical disability with satisfaction with medical care and infrastructure of healthcare facilities among users of medical offices in Peru in 2016. Based on available evidence, we hypothesized that individuals with physical disabilities are less satisfied with medical care and the infrastructure of healthcare centers compared to those without physical disabilities.

## METHODS

### Study design and population

We conducted an analytical cross-sectional study using secondary data from the 2016 National Health User Satisfaction Survey (ENSUSALUD). The study population included users of medical offices in healthcare facilities under the Ministry of Health (MINSA), regional governments, Social Health Insurance (EsSalud), Armed Forces and Police Sanitation (FFAA and PNP), or private establishments.^( 14 )^

### Source of data

The ENSUSALUD 2016 was conducted by the National Superintendence of Health (SUSALUD) in collaboration with the National Institute of Statistics and Informatics (INEI) of Peru. Its primary objective was to assess the level of satisfaction among healthcare service users, as well as healthcare and administrative professionals in Peru.^( 14 )^

The sample size for ENSUSALUD 2016 was determined based on the results of ENSUSALUD 2015. Considering a healthcare services dissatisfaction rate of 26.3%, with a 95% confidence level, and a margin of error between 2 and 5 percentage points, a sample of 13,814 users across 184 healthcare facilities in Peru was estimated. A probabilistic, two-staged, stratified, and independent sampling technique was used for each region of the country. The first sampling unit consisted of healthcare facilities, which were randomly, systematically, and proportionately selected based on the number of visits. The second unit included users who visited the medical offices. This approach ensured national and regional representativeness. Users were surveyed upon leaving the medical office and were asked about their satisfaction levels, health insurance status, general health condition, and any difficulties encountered during medical care. Data was collected from May 13, 2016, to July 9, 2016. Further details on the methodology can be found in the final report of ENSUSALUD 2016.^( 14 )^

### Eligibility criteria

For this analysis, we included the data of male and female Peruvian health facility users aged ≥15 years. Participants with incomplete or inconsistent data on the variables of interest were excluded.

### Variables

#### Satisfaction measures

Satisfaction with medical care and infrastructure of the healthcare facility were assessed as quantitative variables using the questions, “How would you rate the service provided at this health facility today, in terms of…?” which included 10 aspects related to the medical care received, and “How would you rate this health facility in terms of…?” which addressed nine aspects related to the facility’s infrastructure, respectively. Each question was rated on a 10-point scale, with 10 representing the highest level of perceived satisfaction. The total satisfaction scores for medical care and healthcare facility infrastructure were calculated by summing the scores from each aspect ( Table 1 ).

**Table 1:** Questions used to measure satisfaction with medical care and with the health facility infrastructure

How would you rate the service provided at this health facility today, in terms of?											
1	The time it took from when you made the appointment to the date of the consultation?	1	2	3	4	5	6	7	8	9	10
2	The information provided about your health status?	1	2	3	4	5	6	7	8	9	10
3	Compliance with the schedule of medical attention?	1	2	3	4	5	6	7	8	9	10
4	Attention of the administrative staff?	1	2	3	4	5	6	7	8	9	10
5	Treatment by non-medical care staff?	1	2	3	4	5	6	7	8	9	10
6	Treatment by the medical staff?	1	2	3	4	5	6	7	8	9	10
7	Length of consultation?	1	2	3	4	5	6	7	8	9	10
8	Waiting time for care?	1	2	3	4	5	6	7	8	9	10
9	Administrative procedures?	1	2	3	4	5	6	7	8	9	10
10	The clarity with which the treatment and guidelines were explained to you?	1	2	3	4	5	6	7	8	9	10
**How would you rate this health facility in terms of?**											
1	Location?	1	2	3	4	5	6	7	8	9	10
2	Comfort and convenience of the environments?	1	2	3	4	5	6	7	8	9	10
3	Accessibility to the environments?	1	2	3	4	5	6	7	8	9	10
4	Seating or waiting area?	1	2	3	4	5	6	7	8	9	10
5	Room signage and orientation signs?	1	2	3	4	5	6	7	8	9	10
6	Cleanliness and hygiene?	1	2	3	4	5	6	7	8	9	10
7	Privacy of care?	1	2	3	4	5	6	7	8	9	10
8	Health infrastructure?	1	2	3	4	5	6	7	8	9	10
9	Equipment?	1	2	3	4	5	6	7	8	9	10

Score where 1 is bad and 10 is good.

#### Physical disability assessment

The physical disability condition was measured as a nominal qualitative variable using the question, “Regarding your mobility: a) I have no problems walking, b) I have some problems walking, and c) I have to be in bed.” For analysis purposes, responses were categorized as “no physical disability,” “moderate physical disability,” and “severe physical disability.” This classification had been employed in a similar study to assess disability status.^( 15 )^

#### Covariates

We included sociodemographic variables, such as sex (male and female), age groups (15-29, 30-44, 45-59, and ≥60 years), educational level (no education/basic, primary, secondary, and higher), monthly salary in soles (PEN) (<1000, 1000-3000, and >3000), origin (coastal region, highland region, jungle region, and Metropolitan Lima), and health institution (MINSA, EsSalud, FFAA, and PNP and private establishments). Likewise, variables related to user satisfaction were included, such as the language, “what is the language in which you communicate at home” (Spanish, Quechua, or others);^( 16 )^ chronic illness, “do you suffer from a chronic illness or disease?” (no and yes);^( 17 )^ doctor’s explanation, “did the doctor explain to you the illness, problem, or health condition you have?” (yes and no);^( 18 )^ scheduled appointment, “was the appointment or turn for this healthcare given to you today?” (no and yes);^( 19 )^ and waiting time at clinic “time from arrival to health care to meeting the physician” (hours).^( 6 )^

## Statistical processing and analysis

The database was downloaded from the SUSALUD website (http://portal.susalud.gob.pe/blog/base-de-datos-2016/). Statistical analyses were conducted using Stata/MP, version 16.0. The normality of the dependent variables was assessed through visual inspection of histograms and the Shapiro-Wilk test. All analyses accounted for the complex sampling design of ENSUSALUD 2016.

Descriptive results are presented as frequencies and percentages for qualitative variables and as means with standard errors (SEs) for quantitative variables. The Wald test was used to evaluate differences in satisfaction scores. To estimate the primary association between physical disability and satisfaction, multiple linear regression analysis was performed, reporting β coefficients with their corresponding 95% confidence intervals (95%CI). Crude models were initially developed, and variables with a p<0.05 were included in the adjusted models. Collinearity among variables in the adjusted models was assessed by manually calculating the variance inflation factor (VIF),^( 20 )^ with a VIF >3 considered indicative of problematic collinearity. The VIF values were all below 1.5, indicating that collinearity was not a concern in the adjusted model.

## Ethics approval

The study project was approved by the Institutional Research Ethics Committee of the Universidad Científica del Sur (Code 624-2020-PRE15). The ENSUSALUD 2016 data are open-access and available for download from the SUSALUD website. The database does not contain any information that could identify participants.

## RESULTS

**Figure 1:**
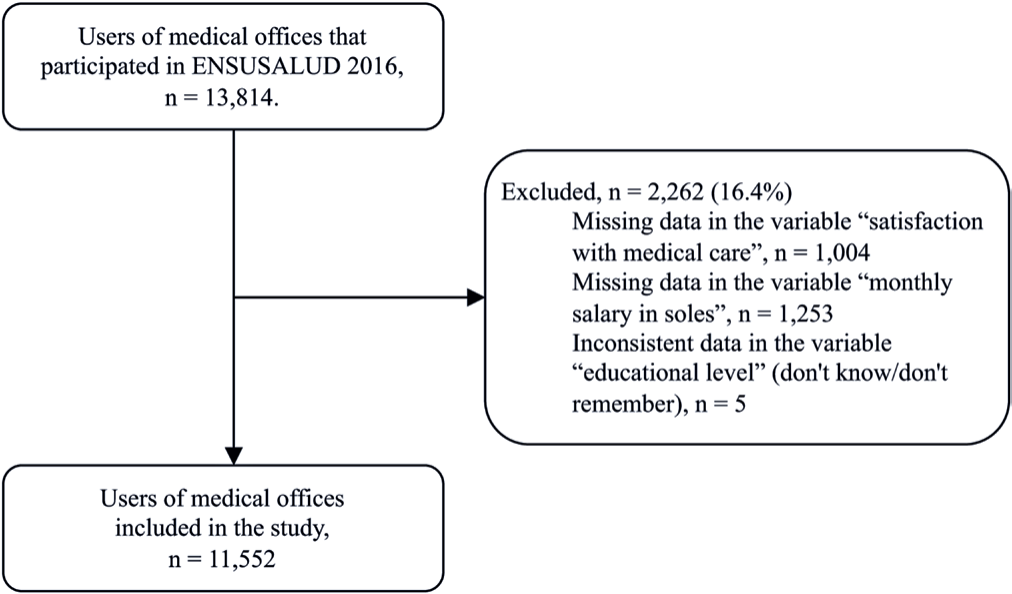
Flow chart for study participant selection

The ENSUSALUD 2016 database included records for 13,814 individuals. A total of 2,262 participants were excluded due to missing or inconsistent data in the variables of interest, resulting in a final sample of 11,552 participants ( Figure 1 ).

Most participants were women (59.0%), aged 15-29 years (29.6%), had completed high school education (41.9%), resided in Metropolitan Lima (42.0%), and reported a monthly income between 1,000 and 3,000 soles (46.6%). Additionally, 46.4% of participants received care at MINSA institutions, 97.8% frequently spoke Spanish, and 52.4% reported having a chronic disease. Most participants (92.9%) received an explanation about their condition from the treating physician, 51.3% had scheduled their appointment in advance, and the average waiting time for care was 1.63 h (SE: 0.24).

Regarding disability status, 22.3% (n=2,614) of participants had moderate physical disability and 0.8% (n=53) had severe physical disability ( Table 2 ). The mean satisfaction score for medical care and healthcare facility infrastructure were 71.15 (SE: 0.23) and 63.15 (SE: 0.21) points, respectively.

**Table 2:** Sample characteristics and differences in satisfaction scores with medical care and health facility infrastructure in users of Peruvian medical offices attended in 2016

Variables	Total (n=11,552) n (%)	Satisfaction with medical care		Satisfaction with the infrastructure	
		Mean (SE)	p value*	Mean (SE)	p value*
Sex			<0.001		<0.001
Male	4,625 (41.0)	72.42 (0.35)		64.19 (0.32)	
Female	6,927 (59.0)	70.28 (0.30)		62.43 (0.27)	
Age groups			<0.001		<0.001
15-29	3,174 (29.6)	70.12 (0.48)		62.23 (0.42)	
30-44	3,310 (27.7)	70.51 (0.42)		62.41 (0.37)	
45-59	2,826 (23.9)	72.29 (0.47)		64.00 (0.43)	
≥60	2,242 (18.8)	72.29 (0.42)		64.63 (0.43)	
Educational level			<0.001		<0.001
No education/basic	244 (1.7)	68.40 (1.23)		60.36 (1.30)	
Primary	1,795 (13.0)	70.48 (0.50)		61.63 (0.47)	
Secondary	4,519 (41.9)	70.19 (0.37)		62.37 (0.34)	
Higher	4,995 (13.4)	72.40 (0.36)		64.48 (0.31)	
Origin			<0.001		<0.001
Coastal region	3,562 (25.3)	68.80 (0.32)		59.54 (0.30)	
Highland region	4,654 (23.6)	69.27 (0.30)		60.52 (0.31)	
Jungle region	2,259 (9.1)	69.96 (0.44)		60.34 (0.41)	
Metropolitan Lima	1,077 (42.0)	73.89 (0.47)		67.43 (0.39)	
Monthly salary (PEN)			<0.001		<0.001
<1000	4,916 (40.9)	68.49 (0.35)		60.73 (0.34)	
1000-3000	5,667 (46.6)	71.55 (0.33)		63.51 (0.29)	
>3000	969 (12.5)	78.40 (0.65)		69.74 (0.59)	
Health institution			<0.001		<0.001
MINSA	5,516 (46.4)	67.39 (0.30)		58.78 (0.29)	
EsSalud	5,098 (32.4)	69.70 (0.33)		63.57 (0.29)	
FFAA and PNP	431 (8.1)	79.45 (1.10)		67.44 (0.92)	
Private establishments	507 (13.1)	82.94 (0.53)		74.96 (0.41)	
Ordinary language			0.252		0.031
Spanish	11,207 (97.8)	71.19 (0.23)		63.23 (0.21)	
Quechua	309 (1.8)	68.63 (0.92)		58.44 (1.04)	
Others	36 (0.4)	72.65 (2.61)		65.18 (2.18	
Chronic illness			0.556		0.343
No	5,406 (47.6)	71.30 (0.36)		63.36 (0.31)	
Yes	6,146 (52.4)	71.03 (0.29)		62.96 (0.28)	
Doctor’s explanation			<0.001		0.001
Yes	10,624 (92.9)	71.91 (0.23)		63.36 (0.22)	
No	928 (7.1)	61.17 (1.01)		60.49 (0.82)	
Scheduled appointment			0.145		<0.001
No	6,395 (48.7)	71.48 (0.33)		61.81 (0.30)	
Yes	5,157 (51.3)	70.81 (0.32)		64.57 (0.28)	
Waiting time at clinic (hours), mean (SE)	1.63 (0.24)	-0.17†	<0.001	-0.17 †	<0.001
Physical disability			0.002		0.005
No physical disability	8,885 (76.9)	71.58 (0.26)		63.48 (0.24)	
Moderate physical disability	2,614 (22.3)	69.69 (0.48)		62.14 (0.46)	
Severe physical disability	53 (0.8)	71.16 (2.74)		60.43 (2.47)	

* Wald test; ^†^ Pearson correlation coefficient.

SE: standard error, MINSA: Ministry of Health, EsSalud: Social Security, FFAA and PNP: Armed Forces and Police Sanitation.

All results are weighted according to the ENSUSALUD 2016 complex sampling.

1 USD=3.3335 PEN in May 2016.

The mean satisfaction scores for medical care and healthcare facility infrastructure were significantly lower among users with moderate (69.69 points and 62.14 points, respectively) and severe (71.16 points and 60.43 points, respectively) physical disabilities compared to those without physical disabilities (71.58 and 63.48 points, respectively) (p=0.002 and p=0.005, respectively).

In the final models for both dependent variables, after adjusting for sex, age group, educational level, region of residence, monthly income, type of healthcare institution, primary language, physician explanation, scheduled appointment, and waiting time at clinic, we found that users with moderate physical disabilities had, averagely 1.96 fewer satisfaction points with medical care (95%CI=-2.95 to -0.97) compared to those without physical disabilities. Similarly, users with moderate and severe physical disabilities had, averagely, 1.86 (95%CI=-2.75 to -0.96) and 6.40 (95%CI=-9.58 to -3.22) fewer satisfaction points, respectively, with the healthcare facility infrastructure compared to users without physical disabilities who attended any healthcare facility in Peru in 2016 ( Table 3 and Tables 1S and 2S, Supplementary Material).

**Table 3:** Association between the condition of physical disability and satisfaction with medical care and with the infrastructure of the health facility in users of medical offices in Peru attended in 2016

Variables	Crude model		Adjusted model*	
	β (95%CI)	p value	β (95% CI)	p value
Satisfaction with medical care				
No physical disability	Reference		Reference	
Moderate physical disability	-1.89 (-2.96; -0.81)	0.001	-1.96 (-2.95; -0.97)	<0.001
Severe physical disability	-0.42 (-5.82; 4.98)	0.880	-1.33 (-6.14; 3.47)	0.586
Satisfaction with the infrastructure				
No physical disability	Reference		Reference	
Moderate physical disability	-1.34 (-2.35; -0.32)	0.010	-1.96 (-2.85; -1.06)	<0.001
Severe physical disability	-3.04 (-7.90; 1.82)	0.220	-6.44 (-9.61; -3.27)	<0.001

* Adjusted for sex, age groups, educational level, origin, monthly salary, health institution, ordinary language, doctor’s explanation, scheduled appointment and waiting time at clinic.

All results are weighted according to the ENSUSALUD 2016 complex sampling.All results are weighted according to the ENSUSALUD 2016 complex sampling.

## DISCUSSION

We confirmed the hypothesis that users with physical disabilities would report lower satisfaction with medical care and the infrastructure of the healthcare facilities they attended. Factors such as perceived discrimination, health status, and physical barriers may contribute to reduced levels of satisfaction. After adjusting for multiple confounding variables, satisfaction scores for medical care were lower among individuals with moderate physical disabilities. Additionally, satisfaction with healthcare facility infrastructure was lower among individuals with both moderate and severe physical disabilities. These findings align with those reported in previous studies.

Satisfaction with medical care was lower among individuals with moderate physical disabilities but not among those with severe physical disabilities. Several studies have used data from the Medicare Current Beneficiary Survey to examine the association between disability status and satisfaction with medical care. One study, which analyzed data from 2001 to 2011 involving 9,323 Medicare beneficiaries under the age of 65, found that individuals with greater limitations in activities of daily living were less likely to be satisfied with access to medical care. However, these limitations were not associated with satisfaction regarding the quality of care received.^( 12 )^ Another study, conducted during the same period which also used Medicare data albeit focusing on individuals aged >65 years, found that the odds of satisfaction with medical care decreased as disability levels increased. However, this association was not significant at higher levels of disability, which is consistent with our findings. The authors suggested that individuals with severe limitations often relied on caregivers to respond on their behalf, potentially leading to responses that did not accurately reflect the individuals’ perceived dissatisfaction.^( 13 )^

Another study involving 16,403 Medicare beneficiaries found that while most individuals with disabilities were satisfied with their care, dissatisfaction increased with the severity of disability. However, this was observed in only 10% of individuals with disabilities.^( 21 )^ A separate study using the same database reported similar findings.^( 22 )^ Likewise, a recent study involving 353,523 individuals from 35 European countries found that those with health vulnerabilities – particularly individuals experiencing difficulties in performing daily activities due to illness, disability, disease, or mental health issues – were less satisfied with their countries’ healthcare systems.^( 23 )^

The existing evidence supports our findings, indicating that disability negatively impacts satisfaction with medical care. One potential factor contributing to this outcome, which has been insufficiently documented, is the perception of discrimination. Discrimination has been associated with a higher likelihood of medical care avoidance among adults with physical disabilities.^( 24 )^ Younger age, higher educational attainment, and unemployment are associated with increased discrimination against individuals with disabilities.^( 25 )^ Stigmatization and prejudice are significant contributors to discrimination within healthcare settings.^( 26 )^ Implicit biases among healthcare professionals result in adverse outcomes and substandard care for individuals with disabilities.^( 27 )^ Therefore, it can be concluded that perceived discrimination based on disability status may also influence satisfaction levels. Another possible explanation could be the health conditions and multiple comorbidities commonly experienced by individuals with disabilities,^( 28 )^ who often expect to receive medical care from specialists with expertise in managing disabling diseases or conditions.^( 29 )^ The lack of training in the care of individuals with disabilities among healthcare professionals contributes to health disparities, creating barriers to high-quality medical care and significantly affecting patient satisfaction.^( 30 , 31 )^ Health systems must adopt inclusive practices, and as part of this transformation, healthcare professionals should receive specialized training on disability-related issues to enhance care quality and improve the quality of life of this vulnerable population.

Satisfaction with healthcare facility infrastructure was lower among individuals with moderate and severe physical disabilities. A scoping review of 96 studies identified numerous barriers to accessing healthcare services for individuals with disabilities, highlighting infrastructure-related obstacles such as physical barriers, poor building accessibility, location of health units, and difficulties in transporting wheelchairs. These barriers were noted from the perspectives of users or caregivers and service providers.^( 32 )^ A qualitative study involving 25 individuals with various disabilities from rural areas further identified structural barriers to healthcare access, including challenges related to transportation and the distance that individuals with lower limb limitations must travel. Additional obstacles included narrow corridors that hinder movement – particularly for wheelchair users – and restrooms that are not adequately accessible for individuals with disabilities.^( 33 )^ Such structural barriers in healthcare facilities limit access to medical care, negatively impacting the health of individuals with disabilities. This likely contributes to the lower satisfaction scores observed, especially among those with severe physical disabilities.

### Public health implications

The results of this study reveal lower satisfaction with both medical care and healthcare facility infrastructure among individuals with physical disabilities. This reduced satisfaction could discourage attendance at healthcare facilities, particularly when this vulnerable group requires specialized medical care. This finding is critical, as individuals with disabilities experience higher morbidity rates,^( 34 )^ more frequent use of emergency services,^( 35 )^ and an increased likelihood of chronic diseases^( 36 )^ compared to those without disabilities. Peru has a General Law for Persons with Disabilities,^( 37 )^ which “guarantees access to comprehensive, quality healthcare services implemented with appropriate infrastructure, equipment, and trained human resources.” However, our findings suggest that simply “guaranteeing” access is insufficient. Healthcare services must also meet quality standards that ensure acceptable satisfaction levels, particularly for the most vulnerable populations.

### Limitations and strengths

This study has some limitations. First, the measurement of physical disability and satisfaction may be subject to social desirability and recall biases, potentially leading to overestimated values. Second, certain variables that could further explain satisfaction levels among healthcare service users were not available in the dataset analyzed. Third, physical disability was assessed using a single, self-reported question that was not specifically designed for this purpose, which may limit the accuracy of the measurement. However, similar questions have been widely used in population-based surveys to assess disability. Fourth, satisfaction was not measured using a validated scale, which may affect the comparability of results with other studies; therefore, comparisons should be made with caution. Fifth, a low frequency of individuals with severe physical disabilities was observed; therefore, results and comparisons involving this group should be interpreted with discretion. Sixth, due to the study’s cross-sectional design, causality between the main variables cannot be established.

As a strength, the ENSUSALUD 2016 was a population-based survey, allowing the results to be generalizable to medical office users across Peru.

## CONCLUSION

In Peru, individuals with physical disabilities who sought care at healthcare facilities in 2016 reported lower levels of satisfaction with medical care and the infrastructure of healthcare facilities compared to those without physical disabilities. Health policymakers must develop strategies to improve the quality of healthcare services, ensuring that vulnerable populations experience higher satisfaction levels, which is essential for promoting continuous healthcare engagement. Additionally, population-based health satisfaction surveys should be more inclusive and incorporate questions specifically designed to assess disability, especially considering that individuals with disabilities are among the groups most affected by limited access to healthcare.

## SUPPLEMENTARY MATERIAL

Teodoro Gaspar-Roman, J. Jhonnel Alarco

**Table 1S:** Multivariate analysis performed to estimate the association between physical disability status and satisfaction with medical care in users of medical offices in Peru attended in 2016 (n=11,552)

Variables	Crude model		Adjusted model*	
	β (95% CI)	p value	β (95% CI)	p value
Physical disability				
No physical disability	Reference		Reference	
Moderate physical disability	-1.89 (-2.96; -0.81)	0.001	-1.96 (-2.95; -0.97)	<0.001
Severe physical disability	-0.42 (-5.82; 4.98)	0.880	-1.33 (-6.14; 3.47)	0.586
Sex				
Male	Reference		Reference	
Female	-2.14 (-3.04; -1.23)	<0.001	-0.90 (-1.70; -0.10)	0.027
Age groups				
15-29	Reference		Reference	
30-44	0.40 (-0.86; 1.65)	0.534	0.54 (-0.53; 1.16)	0.324
45-59	2.17 (0.85; 3.49)	0.001	1.61 (0.46; 2.75)	0.006
≥60	2.41 (0.90; 3.43)	0.001	2.47 (1.20; 3.74)	<0.001
Educational level				
No education/basic	Reference		Reference	
Primary	2.08 (-0.53; 4.69)	0.118	0.88 (-1.67; 3.34)	0.499
Secondary	1.79 (-0.73; 4.31)	0.164	-0.64 (-3.21; 1.94)	0.184
Higher	4.00 (1.49; 6.51)	0.002	-2.08 (-4.70; 0.55)	0.121
Origin				
Coastal region	Reference		Reference	
Highland region	0.46 (-0.39; 1.32)	0.286	-0.42 (-1.22; 0.37)	0.300
Jungle region	1.16 (0.09; 2.23)	0.034	1.80 (0.75; 2.84)	0.001
Metropolitan Lima	5.09 (3.99; 6.19)	<0.001	3.17 (2.13; 4.20)	<0.001
Monthly salary (PEN)				
<1000	Reference		Reference	
1000-3000	3.06 (2.12; 4.00)	<0.001	0.82 (-0.16; 1.79)	0.101
>3000	9.91 (8.47; 11.34)	<0.001	2.36 (0.80; 3.92)	0.003
Health institution				
MINSA	Reference		Reference	
EsSalud	2.31 (1.44; 3.19)	<0.001	2.32 (1.24; 3.41)	<0.001
FFAA and PNP	12.06 (9.83; 14.29)	<0.001	610.23 (8.03; 12.43)	<0.001
Private establishments	15.54 (14.35; 16.74)	<0.001	13.12 (11.64; 14.60)	<0.001
Ordinary language				
Spanish	Reference		Reference	
Quechua	-2.56 (-4.43; -0.70)	0.007	0.99 (-1.86; 2.06)	0.921
Others	1.45 (-3.68; 6.59)	0.579	1.71 (-6.22; 9.63)	0.673
Chronic illness				
No	Reference			
Yes	-0.27 (-1.18; 0.64)	0.559	–	–
Doctor’s explanation				
Yes	Reference		Reference	
No	-10.75 (-12.77; -8.73)	<0.001	-9.94 (-11.92; -7.97)	<0.001
Scheduled appointment				
No	Reference		Reference	
Yes	-0.67 (-1.57; 0.23)	0.145	1.85 (0.90; 2.81)	<0.001
Waiting time at clinic (hours). mean (SE)	-1.62 (-1.98; -1.25)	<0.001	-0.76 (-1.08; -0.45)	<0.001

* Adjusted for sex, age groups, educational level, origin, monthly salary, health institution, ordinary language, doctor’s explanation, scheduled appointment and waiting time at clinic.

All results are weighted according to the ENSUSALUD 2016 complex sampling.

SE: standard error; MINSA: Ministry of Health; EsSalud: Social Security; FFAA and PNP: Armed Forces and Police Sanitation.

**Table 2S:** Multivariate analysis conducted to determine the association between physical disability status and satisfaction with health facility infrastructure in users of medical offices in Peru attended in 2016 (n=11,552)

Variables	Crude model		Adjusted model*	
	β (95% CI)	p value	β (95% CI)	p value
Physical disability				
No physical disability	Reference		Reference	
Moderate physical disability	-1.34 (-2.35; -0.32)	0.010	-1.96 (-2.85; -1.06)	<0.001
Severe physical disability	-3.04 (-7.90; 1.82)	0.220	-6.44 (-9.61; -3.27)	<0.001
Sex				
Male	Reference		Reference	
Female	-1.76 (-2.59; -0.93)	<0.001	-0.77 (-1.48; -0.06)	0.033
Age groups				
15-29	Reference		Reference	
30-44	0.18 (-0.92; 1.28)	0.753	-0.18 (-1.14; 0.76)	0.711
45-59	1.76 (0.58; 2.94)	0.003	0.17 (-0.87; 1.21)	0.745
≥60	2.41 (1.23; 3.58)	<0.001	1.19 (0.08; 2.30)	0.036
Educational level				
No education/basic	Reference		Reference	
Primary	1.27 (-1.45; 3.99)	0.361	-0.31 (-2.65; 2.02)	0.792
Secondary	2.01 (-0.64; 4.65)	0.137	-1.58 (-3.91; 0.75)	0.184
Higher	4.12 (1.49; 6.75)	0.002	-3.07 (-5.43; -0.70)	0.011
Origin				
Coastal region	Reference		Reference	
Highland region	0.97 (0.12; 1.82)	0.025	1.05 (0.31; 1.80)	0.005
Jungle region	0.79 (-0.21; 1.80)	0.123	1.44 (0.52; 2.35)	0.002
Metropolitan Lima	7.88 (6.91; 8.85)	<0.001	6.64 (5.72; 7.56)	<0.001
Monthly salary (PEN)				
<1000	Reference		Reference	
1000-3000	2.78 (1.91; 3.65)	<0.001	-0.25 (-1.11; 0.60)	0.565
>3000	9.01 (7.68; 10.33)	<0.001	0.48 (-1.04; 1.79)	0.600
Health institution				
MINSA	Reference		Reference	
EsSalud	4.79 (3.97; 5.60)	<0.001	4.92 (3.98; 5.86)	<0.001
FFAA and PNP	8.66 (6.77; 10.55)	<0.001	6.62 (4.65; 8.58)	<0.001
Private establishments	16.18 (15.19; 17.17)	<0.001	15.27 (14.03; 16.50)	<0.001
Ordinary language				
Spanish	Reference		Reference	
Quechua	-4.79 (-6.87; -2.70)	<0.001	-1.75 (-3.56; 0.53)	0.057
Others	1.95 (-2.34; 6.24)	0.372	4.89 (-1.98; 11.76)	0.163
Chronic illness				
No	Reference			
Yes	-0.34 (-1.22; 0.43)	0.343	-	-
Doctor’s explanation				
Yes	Reference		Reference	
No	-2.87 (-4.53; -1.21)	0.001	-2.05 (-3.56; -0.54)	0.008
Scheduled appointment				
No	Reference		Reference	
Yes	2.77 (1.96; 3.57)	<0.001	1.19 (0.36; 2.03)	0.005
Waiting time at clinic (hours). mean (SE)	-1.47 (-1.71; -1.22)	<0.001	-0.20 (-0.46; 0.05)	0.120

* Adjusted for sex. age groups. educational level. origin. salary. health institution. usual language. physician’s explanation. scheduled appointment and waiting time at clinic.

All results are weighted according to the ENSUSALUD 2016 complex sampling.

SE: standard error; MINSA: Ministry of Health; EsSalud: Social Security; FFAA and PNP: Armed Forces and Police Sanitation.

## DATA AVAILABILITY:

The content is already available.
